# Supplementary material for: Infection with a Recently Discovered Gammaherpesvirus Variant in European Badgers, Meles meles, is Associated with Higher Relative Viral Loads in Blood
Source: Pathogens. 2022 Oct 6;11(10):1154. doi: 10.3390/pathogens11101154 (PMC9606972; doi:10.3390/pathogens11101154)
Supplement: Supplementary file 1 [file pathogens-11-01154-s001.zip › pathogens-1914758-supplementary_proofread/Supplementary materials_06102022.pdf]

Table S1: List of samples with MusGHV-1 DNA variants and host details of tattoo ID, sex, age, natal group, social group and sett visited (obtained from previous capture records [1,2]

Tattoo ID	Sex	Sample type	Variant	Age at 2018	Natal group	Social group	Sett visited
1012	M	Oral swab	Common	13	PO	PO	PO, SH
1232	F	Genital swab	Common	10	BB	MT	TR, MT, BB
1283	M	Genital swab	Common	9	JH	JH	JH
1341	M	Oral swab	Common	8	GOO	HC	GOO, GO, HC, CP, KH
1354	M	Genital swab	Common	10	PO	PO	PO, P, JkBr, SH
1359	F	Oral swab	Common	8	JKBR	JH	JKBR, JH, PV
1372	F	Genital swab	Common	8	PO	PO	PO, P, SH
1378	M	Genital swab	Common	8	JH	JH	JH, P
1379	M	Genital swab	Common	>8	CP	CP	CP, MT, Gates
1383	M	Genital swab	Common	8	GOO	GOO	GOO, GO
1445	F	Oral swab	Common	8	TC	TC	TC
1469	F	Rectal swab	Common	5	TC	TC	TC
1478	F	Oral swab	Common	5	JKBR	JH	JKBR, JH, CHO
1487	M	Genital swab, blood	Common	7	MT	JH	JH, MT, PV, M2
1504	M	Genital swab	Common	4	HH	HH	HH, GAH, CLO
1512	M	Genital swab	Common	4	GOO	RC	GOO, RC
1516	M	Genital swab	Common	?	NC	NC	NC
1520	F	Genital swab	Common	4	JH	JH	JHA, BB
1525	M	Genital swab	Common	4	P	P	P
1549	M	Genital swab	Common	4	CP	CP	CP, Gates
1584	M	Genital swab	Common	3	P	P	P
1592	M	Genital swab	Common	4	M2?	M2	M2, JH
1631	F	Genital swab	Common	3	MT	BB	BB, MT
1635	M	Genital swab	Common	3	CP?	TC	OL1, OLX, M2, HH, TC
1641	M	Genital swab	Common	2	CP	CP	CP, OL1
1642	F	Genital swab	Common	2	CH	CH	CH
1644	M	Genital swab	Common	2	GAH	CLO	CLO, GAH, HH
1647	M	Genital swab	Common	2	GAH	GAH	GAH, CLO
1668	M	Genital swab	Common	2	P	P	P, Hill End
1669	F	Genital swab	Common	2	GAH	FB	FB, GW
1678	F	Genital swab	Common	2	CH	CH	CH
1694	F	Genital swab	Common	2	LS	LS	LS
1696	F	Genital swab	Common	2	MT	MT	MT, TR, BB

1699	M	Genital swab	Common	1	JH	JH	JHA, JH
1713	M	Genital swab	Common	1	GOO	GOO	GOO
1720	M	Genital swab	Common	1	SH	SH	SH
1722	F	Genital swab	Common	1	GAH	GAH	GAH, FB, GW
1739	F	Genital swab	Common	0	CP	CP	CP
1742	F	Genital swab	Common	0	BL	BL	McBr
1744	M	Genital swab	Common	0	SW	SW	SW
1745	M	Genital swab	Common	0	SH	SH	SHO
1753	F	Genital swab, blood	Common	0	CH	CH	CH
1756	M	Genital swab	Common	0	FB	FB	FB, RC
1763	M	Genital swab	Common	0	BB	BB	BB
1776	F	Genital swab	Common	0	Hedge	Hedge	Hedge
1777	M	Genital swab	Common	2	FS Farm	FS Farm	OB
1778	M	Genital swab	Common	0	FS Farm	FS Farm	Road
1780	F	Genital swab	Common	0	P	P	P
1784	M	Genital swab	Common	0	GAH	GAH	Ingrid B
1785	F	Genital swab	Common	0	GAH	GAH	GAH, CLO
1787	M	Genital swab	Common	2	CHO	CHO	CHO
1793	M	Genital swab	Common	0	CHO	CHO	CHO
1234X	F	Genital swab	Common	10	BB	MT	TR, MT, BB, LS, JH
1239F	F	Genital swab	Common	10	PO	P	PO, P
1737X	M	Genital swab	Common	0	MM	MM	MM
1435	F	Genital swab, blood	Coinfection	6	CH	CH	CH, GAH
1498	F	Rectal swab	Coinfection	5	BL	CH	BL, CH
1751	F	Genital swab, blood	Coinfection	0	CH	CH	CH
1755	M	Genital swab, blood	Coinfection	0	CHO	CHO	CHO
1045X	F	Genital swab, blood	Coinfection	13	CHO	CHO	CHO, Ditch, GAH
1330	M	Genital swab	Novel	8	GOO	RC	GOO, GOA, RC, HC
1622	M	Genital swab	Novel	3	CP	RC	CP, RC,OL64, LS
1746	F	Genital swab	Novel	0	CH	CH	CH
1749	F	Genital swab	Novel	0	CHO	CHO	CHO
1750	F	Genital swab, blood	Novel	0	CH	CH	CH
1754	M	Blood	Novel	0	CH	RC	CH, RC, CC, HSBC

Table S2: Ct values of MusGHV-1 genome by variant in 40 blood samples from badgers of previously known identity (Tattoo ID) and age class

Tattoo ID	MusGHV-1 Variant (genital swab)	MusGHV-1 Variant (oral swab)	MusGHV-1 Variant (Blood)	Age	Age Group	Ct value		
						Spring	Summer	Autumn
1012		Common (spring)		13	Very old		32.56	
1045x	Novel (autumn)			13	Very old	34.30		33.44
1234x	Common (spring, summer)			10	Very old		32.20	34.27
1232	Common (autumn)			10	Very old	34.47		33.99
1283	Common (summer)			9	Very old	36.51	35.90	
1330	Novel (summer)			8	Very old		34.23	
1379	Common (spring)			Unknown	Very old	36.16		33.74
1487	Common (summer)			7	Old	34.97		35.99
1435	Novel (summer)		Dual (summer)	6	Old		31.98	32.36
1478		Common (summer)		5	Old		33.02	
1520	Common (spring)			4	Young	35.31	33.53	
1622	Novel (summer autumn)			3	Young	32.33	30.13	
1694	Common (summer)			2	Young		32.32	33.74
1699	Common (summer)			1	Juvenile		31.88	33.30
1735				0	Juvenile	36.30		
1746	Novel (summer)			0	Juvenile		28.67	
1749	Novel (spring)			0	Juvenile	27.10		
1750	Novel (summer)		Novel (summer)	0	Juvenile		28.74	29.99
1751	Novel (autumn)		Dual (summer)	0	Juvenile	30.77	30.12	30.94
1753	Common (spring)		Common (summer)	0	Juvenile	32.74		
1754			Novel (summer)	0	Juvenile	30.04		
1755	Novel (summer)		Dual (summer)	0	Juvenile	28.87	31.55	31.74
1778	Common (summer)			0	Juvenile		29.97	
1785	Common (summer)			0	Juvenile			30.51

Table S3: Counts of individuals infected with the common or novel MusGHV-1 variant categorized by sex, age class and social groups. There was no significant difference in novel variant prevalence based on sex or age class (Fisher's test: p>0.05); however, individuals infected with the MusGHV-1 novel variant all resided in just 3 (of 25) social groups (CH, CHO, RC), producing a marginally significant spatial pattern.

MusGHV-1 variant	Sex		Age group		Social group																								
	Male	Female	Juvenile	Adult	BB	BL	CH	CH<br>O	CLO	CP	FB	Far<br>m	GA<br>H	GO<br>O	HC	Hed<br>ge	HH	JH	LS	M2	MM	MT	NC	P	PO	RC	SH	SW	TC
Common (*n*)	33	22	18	37	2	1	3	2	1	4	2	2	4	2	1	1	1	7	1	1	1	3	1	5	3	1	2	1	3
Novel (*n*)	4	7	6	5	0	0	5	3	0	0	0	0	0	0	0	0	0	0	0	0	0	0	0	0	0	3	0	0	0
Prevalence of the novel variant	11%	24%	25%	12%	0%	0%	63%	60%	0%	0%	0%	0%	0%	0%	0%	0%	0%	0%	0%	0%	0%	0%	0%	0%	0%	75%	0%	0%	0%
Fisher's test	p=0.1912		p=0.1885		p=0.0518																								

Table S4: List of samples tested for MusGHV-1 in blood and genotyping

Badger ID	Sampling month	Social group	Sex	Age	MusGHV-1 Variant
1045x	Nov-09	CHO	Female	4	Novel+Common
1155	Jun-10	CHO	Female	3	Common
1168	Jun-10	CH	Female	3	Common
1273	Nov-09	CH	Female	0	Common
1332	Jun-10	CHO	Male	0	Common
1335	Jun-10	CHO	Female	0	Novel
1366	Sep-10	CHO	Male	0	Novel
1368	Sep-10	CHO	Female	0	Common
1115	Nov-09	RC	Female	3	Common
1133	Nov-09	RC	Male	2	Common
1165	Nov-09	RC	Male	2	Common
1292	Sep-09	RC	Female	0	Common
1346	Jun-10	RC	Male	0	Common
1342	Jun-10	RC	Female	0	Common

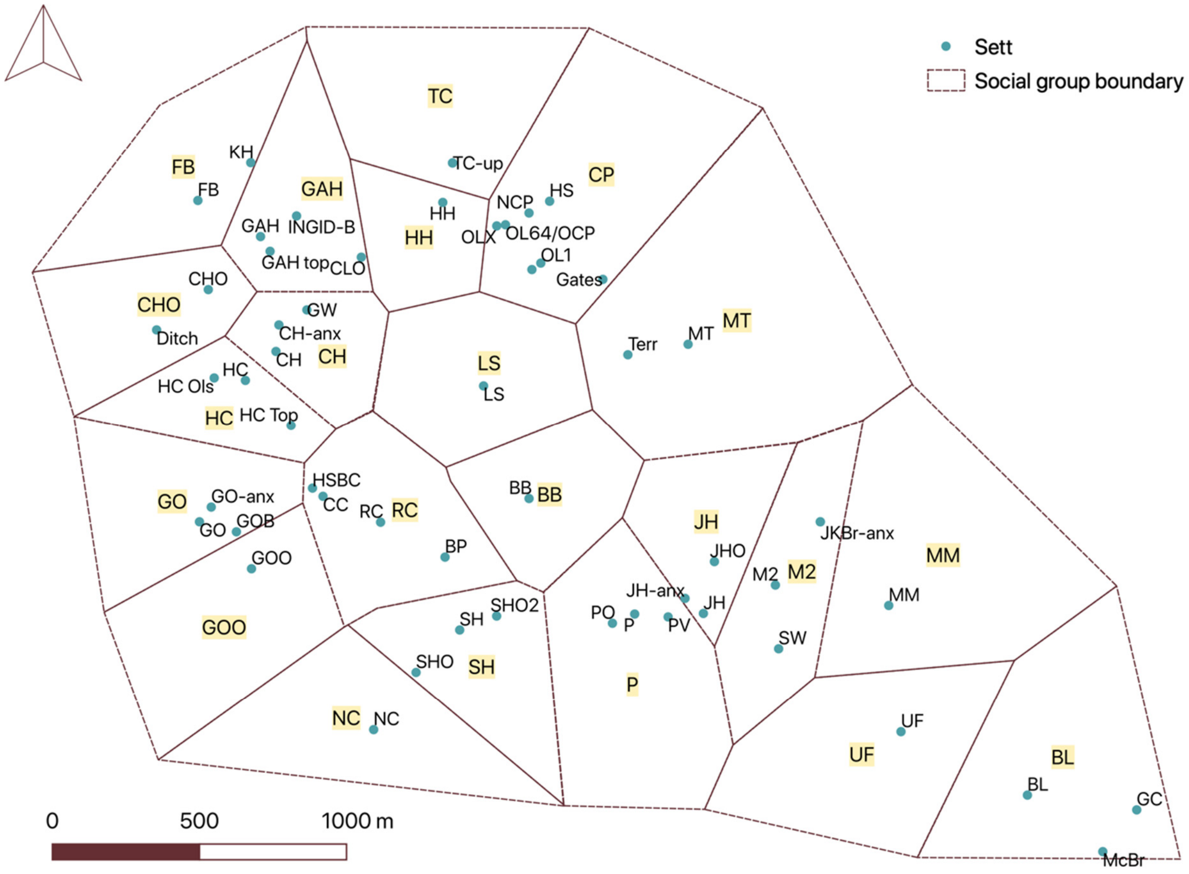

Figure S1: Location of badger setts and range of social groups in Wytham Wood

References:

1. Macdonald, D.W.; Newman, C.; Buesching, C.D. Badgers in the rural landscape—conservation paragon or farmland pariah? Lessons from the Wytham Badger Project. *Wildl. Conserv. Farml. Vol. 2* 2015, *2*, 65–95, doi:10.1093/acprof:oso/9780198745501.003.0004.
2. Sugianto, N.A.; Newman, C.; Macdonald, D.W.; Buesching, C.D. Heterochrony of puberty in the European badger (Meles meles) can be explained by growth rate and group-size: Evidence for two endocrinological phenotypes. *PLoS One* 2019, *14*, 1–21, doi:10.1371/journal.pone.0203910.
